# *Bemisia tabaci* MED Population Density as Affected by Rootstock-Modified Leaf Anatomy and Amino Acid Profiles in Hydroponically Grown Tomato

**DOI:** 10.3389/fpls.2018.00086

**Published:** 2018-02-05

**Authors:** Katja Žanić, Gvozden Dumičić, Marija Mandušić, Gabriela Vuletin Selak, Ivana Bočina, Branimir Urlić, Ivica Ljubenkov, Viljemka Bučević Popović, Smiljana Goreta Ban

**Affiliations:** ^1^Department of Applied Sciences, Institute for Adriatic Crops and Karst Reclamation, Split, Croatia; ^2^Department of Plant Sciences, Institute for Adriatic Crops and Karst Reclamation, Split, Croatia; ^3^Centre of Excellence for Biodiversity and Molecular Plant Breeding (CroP-BioDiv), Zagreb, Croatia; ^4^Department of Biology, Faculty of Science, University of Split, Split, Croatia; ^5^Department of Chemistry, Faculty of Science, University of Split, Split, Croatia; ^6^Department of Agriculture and Nutrition, Institute of Agriculture and Tourism, Poreč, Croatia

**Keywords:** cultivar, GABA, grafting, *Solanum lycopersicum*, spongy parenchyma, tobacco whitefly

## Abstract

*Bemisia tabaci* is one of the most devastating pests in tomato greenhouse production. Insecticide resistance management for *B. tabaci* requires a novel approach that maximizes non-chemical methods for pest control. The aim of this study was to test the effects of rootstocks on *B. tabaci* populations in hydroponically grown tomato plants. In order to contribute to the better understanding of the mechanisms defining the attractiveness of plant to the aerial pest, the effects of rootstocks on leaf anatomy and the amino acid composition of phloem sap were assessed. A two-factorial experimental design was adopted using cultivars (rootstock cultivars and Clarabella) grown as either non-grafted or grafted with cultivar Clarabella as a scion. The rootstock cultivars included Arnold, Buffon, Emperador, and Maxifort. A reduction in *B. tabaci* density was observed using all rootstock cultivars. The number of adult individuals per leaf was 2.7–5.4 times lower on rootstock cultivars than on Clarabella. The number of large nymphs per square centimeter was at least 24% higher on non–grafted Clarabella compared with all other treatments. The leaf lamina thickness and mesophyll thickness were lower in self-grafted Clarabella than in non-grafted or in one grafted on rootstock cultivars; however, the extent of this reduction depended on the rootstock. The leaves with thinner laminae were generally less attractive to *B. tabaci*. Eighteen amino acids were detected in the exudates of phloem sap. In all treatments, the most abundant amino acid was γ-aminobutyric acid (GABA), followed by proline, serine, alanine, and histidine. The scion cultivar Clarabella was the most attractive to *B. tabaci* and had a higher content of leucine than did rootstock cultivars, and a higher content of lysine compared to Buffon and Maxifort. The features modified by rootstock such are changes in leaf anatomy can affect the attractiveness of plants to *B. tabaci*. Thus, the grafting of tomato could constitute a valuable tool in an integrated management strategy against this aerial pest.

## Introduction

The whitefly *Bemisia tabaci* (*Gennadius*) is one of the most devastating horticultural pests throughout the world, particularly in greenhouse crop production. *Bemisia tabaci* exhibits high genetic diversity within the polyphagous species complex. The *B. tabaci* Mediterranean (MED or biotype Q) and Middle East-Asia Minor 1 (MEAM1 or biotype B) species have been recognized as extremely invasive (Frohlich et al., 1999; De Barro et al., 2000).

Vegetable growing in greenhouses is among the most advanced agricultural technologies. Tomato (*Solanum lycopersicum* L.) is an important crop in Croatia as well as throughout the world (FAO, 2014) and is among the crops most often grown in protected cultivation.

*Bemisia tabaci* MED and MEAM1 are among the most serious pests of tomato (Jiao et al., 2012). According to Žanić et al. (2005), Skaljac et al. (2010) and Žanić et al. (2017), only *B. tabaci* MED is widespread in the coastal areas of Croatia.

Applications of insecticides such as neonicotinoids and pyrethroids as well as the insect growth regulator pyriproxyfen remain an important tool for the control of *B. tabaci*. Nevertheless, the excessive use of these insecticides has resulted in the reduction in their efficacy against pest populations due to the development of insecticide resistance (Elbert and Nauen, 2000; Ahmad et al., 2002; Roditakis et al., 2005; Karunker et al., 2008; Horowitz and Ishaaya, 2014; Wang et al., 2017). According to Ellsworth and Martinez-Carrillo (2001), insecticide resistance management for *B. tabaci* involves maximizing each non-chemical tactic of the pest control; these maximizations can contribute to preserving the susceptibilities of these pest populations.

To achieve profitability and contribute to environmental protection, constant improvement of vegetable production technology involves new approaches related to pest management. Thus, vegetable grafting has received more attention in recent years as a sustainable practice for mitigation of abiotic and biotic stresses (Colla et al., 2010; Louws et al., 2010; Savvas et al., 2010; Schwarz et al., 2010). Grafting is a common practice in watermelon and cucumber production in Croatia (Goreta et al., 2008; Goreta Ban et al., 2014), but tomato grafting has been introduced to greenhouse production recently.

While the effects of grafting on soil-borne diseases and nematodes are known, insufficient information is available regarding the effects of grafting on foliar pest populations, e.g., whiteflies (Žanić et al., 2017). According to our previous study (Žanić et al., 2017), tomato grafting onto Arnold and/or He-Man rootstocks can be a useful tool in an integrated management strategy against *B. tabaci*. The study showed that number of *B. tabaci* large nymphs was affected by both nitrogen and rootstock as well as their interaction. Alam et al. (1995) reported that the use of wild *Solanum* sp. as rootstock reduced the density of *B. tabaci*, leading to a reduced occurrence of the symptoms of viruses transmitted by whitefly. Alvarez-Hernandez et al. (2009) and Cortez-Madrigal (2010) found a lower occurrence of *B. tabaci* on tomatoes grafted onto populations of wild *Solanum* sp. than on tomatoes of non-grafted plants.

The mechanisms contributing to plant resistance to specific pests are still a matter of debate. *Bemisia tabaci* is a phloem feeder (van Lenteren and Noldus, 1990; Byrne and Bellows, 1991) and its feeding behavior is linked to stylet penetration through the leaf. Leaf lamina thickness showed a positive correlation with the number of *B. tabaci* adults in different eggplant varieties (Hasanuzzaman et al., 2016). Similar results were found in black gram, and leaf lamina thickness in that crop was positively correlated with the number of *B. tabaci* eggs, nymphs and adults on the infested plants (Taggar and Gill, 2012). The role of leaf anatomy in preventing tomato plant penetration by another important whitefly *Trialeurodes vaporariorum* (Westwood) was proposed by Jauset et al. (2000). The authors suggested the thickness of abaxial tomato epidermis and leaf laminae, in conjunction with nitrogen level and water content, could influence the survival *T. vaporariorum*.

Dietary nitrogen (N) is one of the factors influencing whitefly infestations of agricultural crops (Jauset et al., 1998, 2000; Bi et al., 2001; Athar et al., 2011; Žanić et al., 2011). In phloem sap, N is present mainly in the form of free amino acids, and the sap also contains soluble proteins (Ziegler, 1975). As phloem feeders, *B. tabaci* ingest a diet rich in soluble carbohydrates and relatively high levels of free amino acids (Ziegler, 1975; Buchanan et al., 2000). The influence of amino acids composition and level on life traits of phloem feeders were studied as a consequence of N fertilization or plant age (Blackmer and Byrne, 1999; Crafts-Brandner, 2002; Karley et al., 2002; Bi et al., 2003; Gould et al., 2007). However, there are no data about effect of rootstock or grafting on amino acids composition in tomato, and its relation with *B. tabaci*. The main amino acids translocated by tomato phloem are glutamine and glutamate, followed by aspartate, threonine, and serine (Valle et al., 1998).

The aim of this study was to test the effects of rootstock on *B. tabaci* populations, leaf anatomy and the amino acid composition of phloem sap in hydroponically grown tomato plants. This aim was approached by examining four different rootstock cultivars and Clarabella, either non-grafted or grafted, using Clarabella as a scion.

We hypothesized that rootstock cultivars are carriers of resistance that contributes to the reduction of *B. tabaci* infestation and populations density in hydroponically grown tomato crop. The experiment was set-up to test the relation between *B. tabaci* density and rootstock-modified changes in leaf anatomy and free amino acid profile in phloem sap.

## Materials and methods

### Plant material

**Figure 1:**
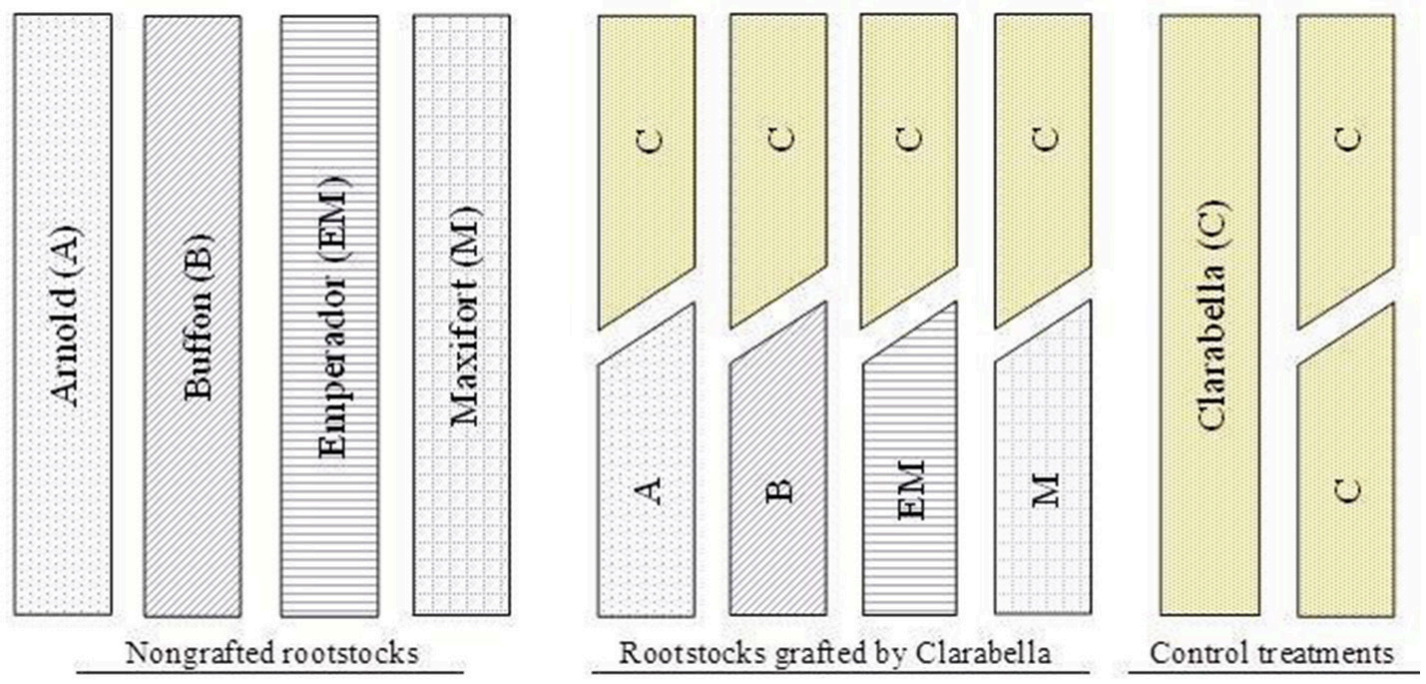
Scheme description of the plant material used in the study including: non-grafted rootstock cultivars Arnold (A), Buffon (B), Emperador (EM), and Maxifort (M) or grafted with scion cultivar Clarabella (C); and non-grafted and self-grafted scion cultivar.

The experiment was conducted in an unheated greenhouse at the Institute for Adriatic Crops at Split (43°30′17.17″N, 16°29′49.71″E) in the Mediterranean area of Croatia. A two-factorial experimental design was adopted in which cultivars (rootstock cultivars and Clarabella) were grown as non-grafted or grafted with cultivar Clarabella as a scion (Figure 1). The rootstock cultivars included Arnold, Buffon (Syngenta Seeds Basel, Switzerland), Emperador (Rijk Zwan, The Netherlands), and Maxifort (De Ruiter, The Netherlands), while the scion cultivar Clarabella (Rijk Zwan, The Netherlands) was grown as a non-grafted or self-grafted scion (Figure 1). This experiment was conducted on young plants in accordance with the goal of the study and did not include harvest.

### *Bemisia tabaci* culture

*Bemisia tabaci* MED adult insects were collected from the insectary of the institute, where they had been maintained on cotton seedlings (*Gossypium hirsutum* L. cv. Acala) in a growth chamber under a 14:10 h light/dark photocycle at 26°C and 60% relative humidity (RH). This population was used to infest peppermint plants (*Mentha piperita* L.) as a host plant favored by *B. tabaci*. Whitefly-infested peppermint plants were kept in a low tunnel covered by non-woven, polypropylene material (Novagryl P30; Fiberweb France, A Berry Plastics Company, France) inside the greenhouse under conditions that were favorable for pest development (Žanić et al., 2011). Uniformly infested peppermint plants were further used for the infestation of tomatoes.

### Plant treatments and cultivation

The seeds of tomato rootstocks and scion were sown in an organic substrate (Brill Type 4; Gebr. Brill Substrate GmbH & Co. KG, Germany). Splice grafting was performed 1 month after sowing (21 September; Table 1) at the lower epicotyl as recommended for solanaceous crops and according to Lee et al. (2010). The seedlings of rootstock and scion were cut completely across and diagonally. Their end surfaces were overlapped in a way the cambiums are in contact, and secured using a silicone grafting clip, sized for stem diameters of 2.0 mm (Bato Plastics B.V., The Netherlands). Grafted seedlings were cultivated under reduced light conditions (10% of daily light intensity) at a relative humidity higher than 95% and a temperature from 22 to 25°C for 7 days until callus formed. The grafted and non-grafted seedlings that had two fully developed leaves were planted into rockwool cubes (7.5 × 7.5 × 6.5 cm^3^; KRANIZOL s.r.o., Czech Republic) 10 days after grafting and fertigated with a nutrient solution prepared in accordance with the methods of Sonneveld and Straver (1994); the solution had an electrical conductivity of 3 dS/m and a pH of 5.5.

**Table 1:** Days after sowing (DAS) and dates at which plant cultivation measures and sampling measurements were conducted.

	**Activities**									
	**Plant cultivation**				**Sampling and measurements**					
**Description**	**Sowing**	**Grafting**	**Planting into rockwool cubes**	**Transplanting into rockwool slabs**	**Infestation**	**Counting of adults**	**Counting of eggs**	**Phloem sampling and anatomy measurements**	**Counting of nymphs**	**The end of the experiment**
DAS	0-3	30	40	58	59	61–67	69	79	93	98
Date	22–24 Aug.	21 Sept.	1 Oct.	19 Oct.	20 Oct.	22–29 Oct.	30 Oct.	11 Nov.	24 Nov.	28 Nov.

The experiment was arranged in a randomized block design and consisted of four replications. Grafted and non-grafted young plants with five fully expanded leaves of approximately 22–35 cm in height were transplanted into rockwool slabs (7.5 × 20 × 100 cm^3^) on 19 October 2015 (Table 1). There were four transplants per slab. Each treatment comprised 16 plants and was replicated four times with four plants each. The plants were fertigated with nutrient solution (Sonneveld and Straver, 1994) containing 205 mg/L N and composed of 1.25 mM ${\text{NH}}_{4}^{+}$, 12.71 mM ${\text{NO}}_{3}^{-}$, 1.25 mM H_2_${\text{PO}}_{4}^{-}$, 8.75 mM K^+^, 2 mM Mg^2+^, 4.25 Ca^2+^, 1.75 mM ${\text{SO}}_{4}^{2-}$, 15 μM Fe^2+^, 10 μM Mn^2+^, 5 μM Zn^2+^, 0.5 μM Mo^+^, 0.75 μM Cu^2+^, and 30 μM B^+^. The nutrient solution was applied using a drip irrigation system during a 40-day period. The temperature in the glasshouse recorded at 2 p.m. ranged from 12.5° to 30°C.

The plants were exposed to adults of *B. tabaci* via infested peppermint plants on 20 October 2015 (the plants had 5–7 leaves; Table 1). One highly infested peppermint plant was used per rockwool slab, and this infestation source was removed after 2 days.

### Pest sampling

Sampling of whitefly instars was performed according to the methods of Žanić et al. (2011). The seven oldest leaves of the selected plants were used for pest density assessments.

Assessments of adult numbers were conducted early in the morning (7 a.m.) at 2, 5, and 8 days after infestation (DAI) using the leaf-turn method (Table 1). Adult numbers were assessed on all (5–7) the fully developed leaves of 8 selected young plants in each treatment.

To record the densities of eggs at 10 DAI (Table 1), all leaves were collected from 8 plants per treatment and examined in the laboratory. The total number of eggs per leaf was recorded using a stereo microscope (Stemi 2000, Zeiss, Germany). The leaf area was determined using a leaf area meter LI-3050A (LI-COR, Nebraska, USA), and these data were also expressed as the number of eggs per 100 cm^2^.

The density of the large nymphs (fourth instar to pupae stages) was assessed on 4th, 5th, and 6th leaves of two sampled plants per plot at 35 DAI (24 November; Table 1). The assessment was conducted on the date approximately coinciding with the completion of the pest life cycle. In accordance with the procedure described by Liu (2004), large nymphs were counted on 4 leaf disks (radius = 1 cm), 2 from each side of the middle vein of each leaflet. The total number of individuals on each 3.14-cm^2^ disc was recorded using a stereomicroscope and expressed as the number of nymphs per square centimeter.

### Leaf anatomy measurements

**Figure 2:**
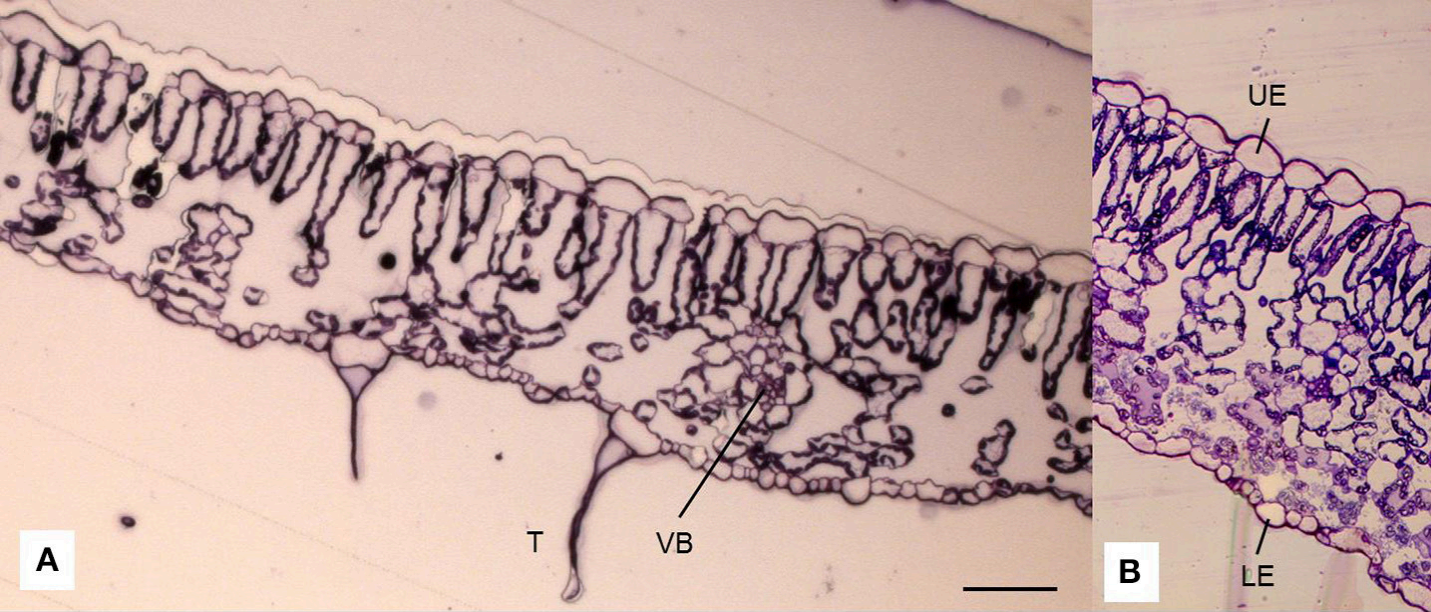
Cross-section of a leaf lamina of tomato (*Solanum lycopersicum* L.) observed under a light microscope (**A**; in addition, detail **B**) showing the epidermis on the adaxial (upper; UE) and abaxial (lower; LE) sides of the leaf, palisade (PM), and spongy mesophyll (SM), trichomes (T) that occur on the epidermis **(A)** and vascular bundles (VB) scattered within spongy parenchyma. Scale bar = 50 μm.

One leaflet of the fifth leaf from the top of each of four plants in each treatment was removed on 11 November (Table 1). Two samples (5 mm^2^) of each leaflet (one from each side of the midrib) were fixed in 3.5% paraformaldehyde in 0.1 M phosphate buffer solution (PBS; pH 7.3) for 24 h at 4°C and then in 3% glutaraldehyde in 0.1 M PBS (pH 7.2) for 2. The tissue was then postfixed in 2% osmium tetroxide for 1 h and then dehydrated in an ascending series of acetone. After dehydration, the tissue samples were embedded in the Durcupan resin (Fluka AG Buchs, Switzerland) and cut transversally (Sheehan and Hrapchak, 1980). At least four semithin 0.5-μm sections per sample were stained with 1% toluidine blue and then examined under a light microscope (Axioskop 2 plus, Carl Zeiss, Jena, Germany) using 400X magnification and Axiovision SE64 software (Carl Zeiss, Jena, Germany). In each section (Figures 2A,B), measurements of different leaf anatomy characteristics were made (the thickness of the lamina, the thickness of the abaxial (lower) and adaxial (upper) layers, and the thickness of the mesophyll). The distance from the underleaf surface to the center of the nearest minor vascular bundle was also measured. The minor vascular bundles were defined according to the methods of Cohen et al. (1996).

### Amino acid extractions from phloem sap

Phloem sap was extracted from plant treatments according to the methods of King and Zeevaart (1974) and Gould et al. (2007) on 11 November (Table 1). The terminal leaflet was excised from a compound leaf that was midway along the shoot axis, after which the excised leaflet was inserted immediately into a 1.5-ml solution of 20 mM ethylenediamine tetraacetic acid (EDTA; pH 7.0) for 2 h in the dark at 25°C. The leaflet samples were then transferred to 1.5-ml vials that contained deionized water for 12 h of sap collection in the dark. The phloem sap samples exuded in diH_2_O for 12 h were used for chemical analysis. The petioles were removed from the vials, and the phloem sap fraction was frozen at −20°C until analysis. The amino acids in the phloem exudates were separated by reverse-phase HPLC (Jones et al., 1981) using a Perkin Elmer Series 200 LC system in conjunction with Waters AccQ•Tag™ derivatization chemistry, columns and fluorescence detection.

### Statistical analysis

The data were tested for normality of distribution and homogeneity of variance and transformed when necessary, after which they were subjected to two-way analysis of variance using proc glm of SAS software (SAS Institute Inc., Cary, New Carolina). When *F*-tests were significant, the means of the main factors and interactions were compared using the least significant difference test at *P* < 0.05.

## Results

### Adult density and oviposition

The effects of rootstock cultivar on density of *B. tabaci* adults were found at 2, 5, and 8 DAI (Table 2), while the effects of grafting were recorded at 2 DAI. The interaction between rootstock cultivar and grafting was not significant. The number of adult individuals per leaf was 2.7–5.4 times lower on rootstock cultivars than on Clarabella, while the difference among rootstock cultivars was not found. The initial assessment at 2 DAI showed that grafted treatments were 36% more attractive to the pests than non-grafted treatments.

**Table 2:** Effects of rootstock cultivar and grafting with Clarabella as a scion on *Bemisia tabaci* adult density and oviposition.

**Treatment**	**Number of adults per leaf**			**An oviposition 10 DAI**		**Leaf area (cm^2^/plant)**
	**2 DAI^a^**	**5 DAI**	**8 DAI**	**Number of eggs per plant**	**Number of eggs per cm^2^**	
**CULTIVAR**						
Arnold	2.86 ± 0.48 b^b^	6.00 ± 0.87 b	6.37 ± 0.78 b	709.5 ± 156.6 b	157.9 ± 45.9 b	535.5 ± 95.9 a
Buffon	1.79 ± 0.27 b	5.46 ± 0.85 b	5.80 ± 0.58 b	651.1 ± 254.4 b	129.7 ± 53.8 b	573.8 ± 110.9 a
Clarabella	9.74 ± 1.28 a	20.29 ± 2.67 a	18.74 ± 1.97 a	2111.0 ± 501.5 a	509.8 ± 149.6 a	477.4 ± 45.3 a
Emperador	2.38 ± 0.40 b	6.10 ± 0.99 b	6.26 ± 0.68 b	896.4 ± 158.5 b	155.7 ± 25.2 b	584.6 ± 63.1a
Maxifort	3.59 ± 0.61 b	4.54 ± 0.67 b	5.36 ± 0.53 b	512.4 ± 127.0 b	115.6 ± 24.3 b	436.3 ± 47.4 a
*F*(*df* = 4)	21.086	21.794	29.361	5.233	4.781	0.69
*P*	<0001	<0001	<0001	0.0026	0.0042	0.6054
**GRAFTING**						
No	3.45 ± 0.48 b	8.02 ± 1.03 a	7.88 ± 0.75 a	1085.3 ± 246.4 a	263.56 ± 67.12 a	438.02 ± 36.3 b
Yes	4.70 ± 0.49 a	8.94 ± 0.88 a	9.14 ± 0.71 a	866.9 ± 172.7 a	163.97 ± 42.22 a	605.03 ± 51.7 a
*F*(*df* = 1)	3.932	0.522	1.773	0.740	2.139	6.83
*P*	0.048	0.4703	0.1836	0.3965	0.1540	0.0145
**C** × **G**						
*F*(*df* = 4)	1.137	0.653	1.048	0.615	0.603	2.08
*P*	0.3385	0.6252	0.3818	0.655	0.6637	0.1109

a *Days after infestation*.

b *Results are expressed as the mean ± SE. For each main effect, different lowercase letters in a column indicate a significant difference by the least significant difference test at P < 0.05*.

The oviposition of *B. tabaci* was influenced by cultivar (Table 2). The effects of grafting as well as cultivar × grafting interactions were not recorded. The number of eggs oviposited on the leaves of cultivar Clarabella was 2.4–4.1 times higher than on leaves of rootstock cultivars at 10 DAI. Consequently, egg density was 3.2–4.4 times higher on Clarabella than on the rootstock cultivars. The leaf area of measured plants was not affected by cultivar but grafted plants had a larger leaf area than non-grafted plants.

### Density of *B. tabaci* large nymphs

The effects of rootstock cultivar and grafting on the density of *B. tabaci* large nymphs were observed at 35 DAI by stereomicroscopic examination of the 4th, 5th, and 6th leaves (Table 3). The number of large nymphs per square centimeter was 2.2–7.9 times lower on rootstock cultivars than on Clarabella at three leaf positions. The density of large nymphs was 30–46% higher on grafted treatments than on non-grafted treatments.

**Table 3:** Effects of rootstock cultivar and grafting with Clarabella as a scion on *B. tabaci* large nymphs density at 35 days after infestation (DAI).

**Treatment**	**No. of large nymphs/cm**^**2**^		
	**4th leaf**	**5th leaf**	**6th leaf**
**CULTIVAR**			
Arnold	2.03 ± 0.16 c^a^	2.49 ± 0.20 b	2.13 ± 0.18 b
Buffon	3.14 ± 0.28 b	2.21 ± 0.18 b	1.20 ± 0.09 d
Clarabella	10.86 ± 0.49 a	11.94 ± 0.73 a	4.59 ± 0.34 a
Emperador	1.38 ± 0.10 c	2.03 ± 0.15 b	1.29 ± 0.12 cd
Maxifort	1.87 ± 0.14 c	2.99 ± 0.26 b	1.80 ± 0.13 bc
*F* (*df* = 4)	163.53	142.51	71.81
*P*	<0.001	<0.001	<0.001
**GRAFTING**			
No	3.35 ± 0.25 b	3.52 ± 0.27 b	1.82 ± 0.15 b
Yes	4.36 ± 0.24 a	5.14 ± 0.32 a	2.59 ± 0.12 a
*F*(*df* = 1)	6.26	17.84	24.33
*P*	0.0125	<0.001	<0.001
**C** × **G**			
*F*(*df* = 4)	8.83	1.24	8.65
*P*	<0.001	0.2901	<0.001

a *Results are expressed as the mean ± SE. For each main effect, different lowercase letters in a column indicate a significant difference by the least significant difference test at P < 0.05 if the interaction was not significant*.

**Figure 3:**
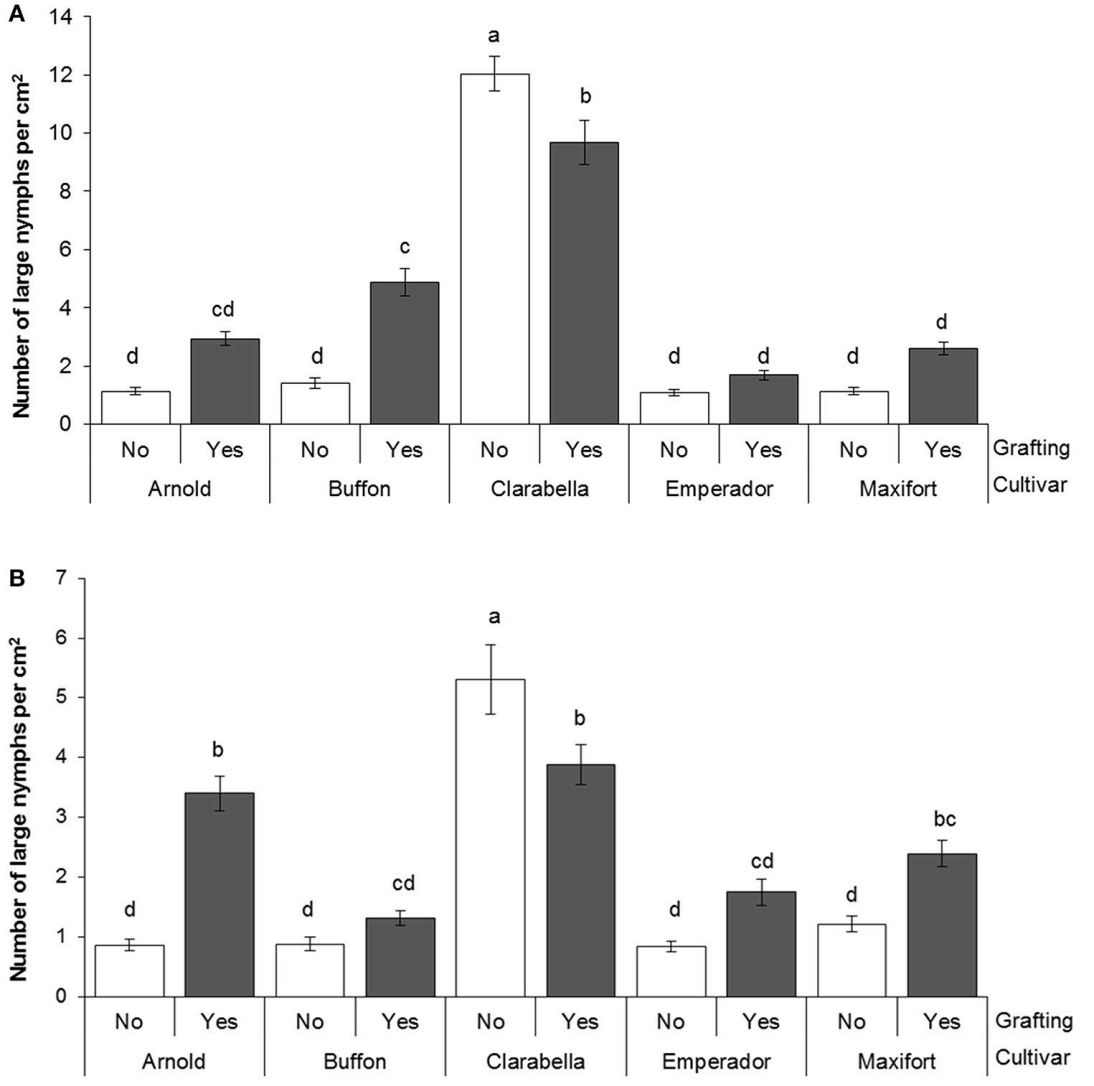
*Bemisia tabaci* large nymph density at 35 days after infestation (DAI) assessed on non-grafted rootstock cultivars or grafted cultivars that used Clarabella as a scion at the 4th leaf position (**A** scale 0–14) and the 6th leaf position (**B** scale 0–7). The results are expressed as the means ± SE. Different lowercase letters indicate significant differences according to the least significant difference test at *P* < 0.05.

The large nymphs density on 4th and 6th leaf was affected by cultivar × grafting interaction (Table 3, Figure 3). On the 4th leaf, non-grafted Clarabella had 24% higher number of large nymphs per square centimeter compared with other treatments (Figure 3A). The number of large nymphs on the 4th leaf of rootstock cultivars Arnold, Emperador, and Maxifort, were similar to observation on non-grafted plants and those grafted using Clarabella as a scion. On the 6th leaf, more than 36% higher density of large nymphs was recorded on non-grafted Clarabella compared with other treatments (Figure 3B). When Buffon, Emperador, and Maxifort were used as rootstock cultivars, the large nymph density was not significantly different between non-grafted plants and those grafted using Clarabella as a scion.

### Leaf anatomy characteristics

**Figure 4:**
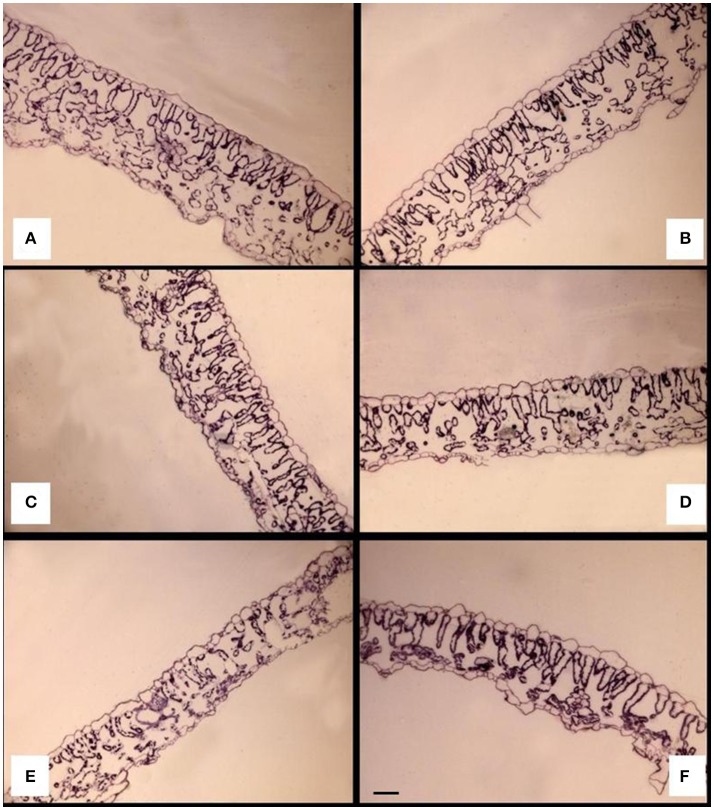
Cross-sections of the leaf laminae of non-grafted Clarabella plants **(A)**; self-grafted Clarabella plants **(B)**; and plants grafted with the cultivar Clarabella as a scion in conjunction with rootstock cultivars Arnold **(C)**, Emperador **(D)**, Buffon **(E)**, and Maxifort **(F)**, observed under a light microscope. Scale bar = 50 μm **(A–F)**.

The adaxial and abaxial epidermal cells were in one layer, and the adaxial cells had a larger volume regardless of treatment (Figures 4A–F). In non-grafted Clarabella, the mesophyll was mostly composed of spongy parenchyma and had large intercellular spaces (Figure 4A). A smaller size of spongy parenchyma was observed for self-grafted Clarabella or Clarabella scion grafted on rootstocks particularly Buffon and Maxifort (Figures 4E,F).

The observed leaf anatomy characteristics important for *B. tabaci* oviposition and feeding ranged as follows: leaf lamina thickness from 101.45 to 171.43 μm, abaxial epidermis thickness from 8.46 to 12.31 μm, and mesophyll thickness from 80.95 to 145.20 μm. All observed leaf anatomy characteristics were significantly affected by cultivar, grafting, and their interaction (Table 4).

**Table 4:** Two-way analysis of variance of the effect of cultivar (Arnold, Buffon, Clarabella, Emperador, and Maxifort) and grafting on tomato leaf anatomy characteristics (Thickness) and Distance from the underleaf surface to the center of the vascular bundles (Distance).

**Variable**	**Cultivar (C)**			**Grafting (G)**			**C** × **G**		
	***df***	***F***	***P***	***df***	***F***	***P***	***df***	***F***	***P***
**THICKNESS**									
Lamina	4	235.18	<0.0001	1	50.79	<0.0001	4	52.51	<0.0001
Mesophyll	4	201.36	<0.0001	1	21.09	<0.0001	4	58.52	<0.0001
Abaxial epidermis	4	12.29	<0.0001	1	6.5	0.0113	4	5.82	0.002
Adaxial epidermis	4	22.35	<0.0001	1	6.12	0.0140	4	17.28	<0.0001
Distance	4	31.24	<0.0001	1	10.68	0.0014	4	10.37	<0.0001

**Figure 5:**
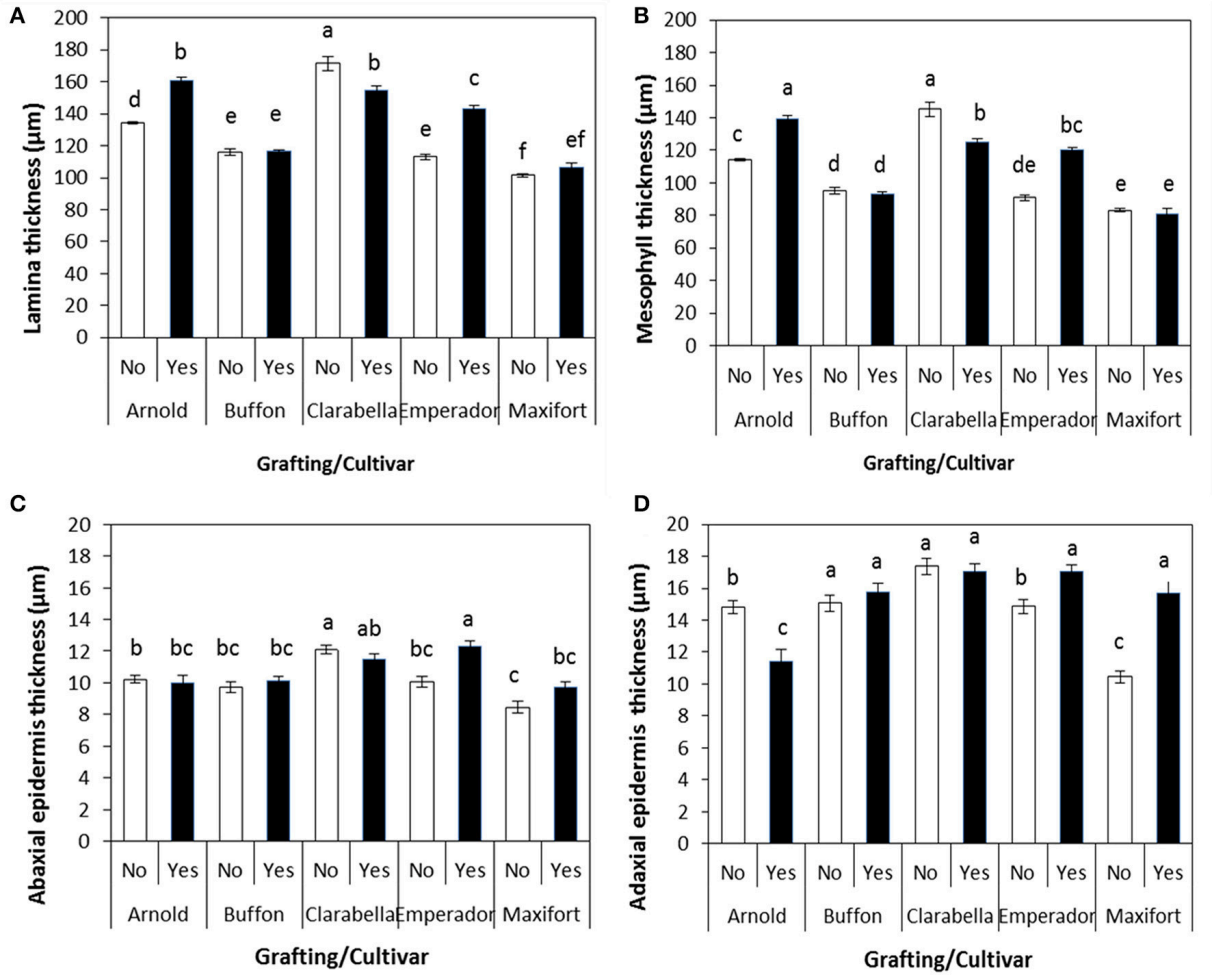
Leaf anatomy characteristics (**A**, lamina thickness; **B**, mesophyll thickness; **C**, abaxial epidermis thickness; and **D**, adaxial epidermis thickness) of non-grafted rootstock cultivars or cultivars grafted using Clarabella as a scion. The results are expressed as the means ± SE. Different lowercase letters indicate significant differences according to the least significant difference test at *P* < 0.05.

**Figure 6:**
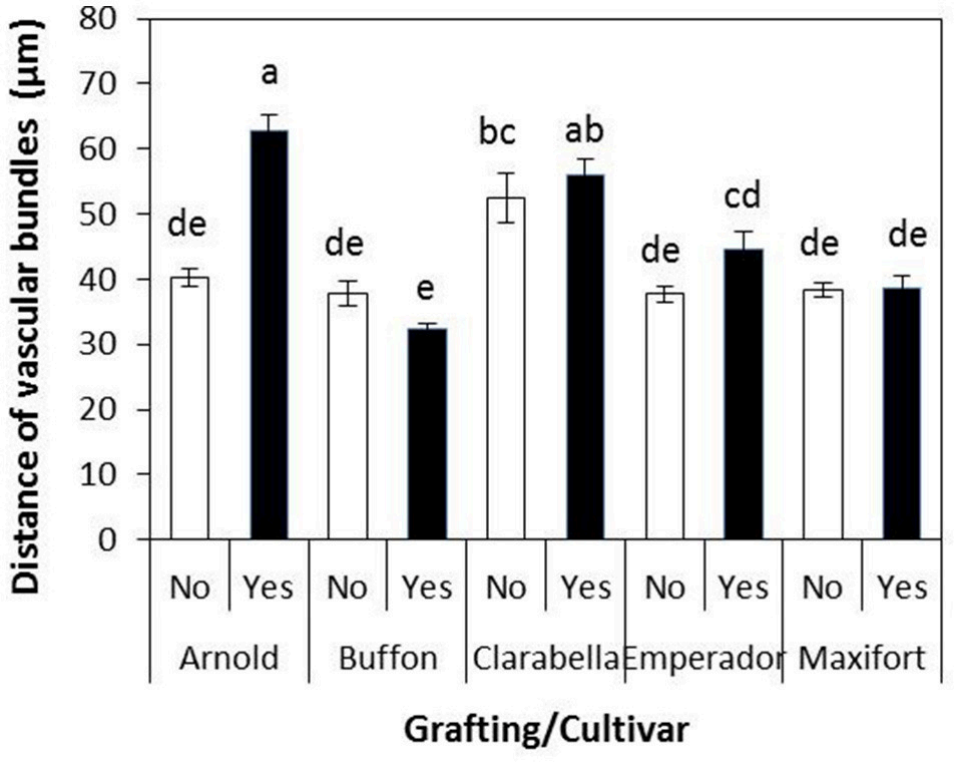
Distance from the underleaf surface to the center of the vascular bundles of non-grafted rootstock cultivars or cultivars grafted using Clarabella as a scion. The results are expressed as the means ± SE. Different lowercase letters indicate significant differences according to the least significant difference test at *P* < 0.05.

The effect of interaction between cultivar and grafting is shown in Figures 5A–D, 6. The thickness of leaf laminae was the highest in non-grafted Clarabella, followed by Arnold grafted using Clarabella as a scion and self-grafted Clarabella (Figure 5A). The thickest mesophyll was found in non-grafted Clarabella, followed by Arnold grafted using Clarabella as a scion and self-grafted Clarabella (Figure 5B). The plants of non-grafted Maxifort rootstock and those grafted using Clarabella as a scion had the thinnest mesophyll. The abaxial epidermis was thickest in Emperador rootstock grafted using Clarabella as a scion as well as in non-grafted Clarabella, followed by self-grafted Clarabella (Figure 5C). The adaxial epidermis was thicker in non-grafted and self-grafted Clarabella plants, non-grafted Buffon plants or those grafted using Clarabella as a scion, and Emperador and Maxifort plants grafted using Clarabella as a scion than in plants of the other treatments (Figure 5D).

The distance from the underleaf surface to the center of the nearest minor vascular bundle was affected by cultivar, grafting, and their interaction (Table 4). An interaction cultivar × grafting is presented in Figure 6. This distance ranged from 32.45 to 62.71 μm; the longest value was recorded in leaves of Clarabella scion grafted onto Arnold rootstock, followed by self-grafted and non-grafted Clarabella plants. In the treatment most preferred by *B. tabaci*, i.e., non-grafted Clarabella, the distance from the underleaf surface to the center of the nearest minor vascular bundle was 52.54 μm.

### Free amino acids in the phloem sap

The amino acid composition of the tomato leaf phloem sap of plants infested with *B. tabaci* is presented in Table 5. Eighteen amino acids were detected in the phloem sap exudates. In all treatments, the most abundant amino acid was γ-aminobutyric acid (GABA), followed by proline, serine, alanine, and histidine. Differences among cultivars were found for GABA, cysteine, lysine, and leucine percentage in the phloem sap, and grafting reduced only the serine percentage in the phloem sap. Interactions between cultivar and grafting were not detected (Table 5). The GABA percentage in the phloem sap was 10–13% lower in Maxifort than in Arnold, Clarabella and Emperador, while the percentage of cysteine was 45.5 and 54.5% lower in Arnold and Buffon than in Emperador, respectively. Compared with other cultivars, the pest-favored cultivar Clarabella had the highest percentage of leucine and 19–28% higher percentage of lysine than did the rootstock cultivars Buffon and Maxifort, respectively.

**Table 5:** Effects of rootstock cultivar and grafting with Clarabella as a scion on the free amino acid composition (%) in the phloem sap of tomato plants at 21 days after infestation.

**Amino acid**	**Rootstock (R)**					**Grafting (G)**		**Significance**		
	**Arnold**	**Buffon**	**Clarabella**	**Emperador**	**Maxifort**	**No**	**Yes**	**R**	**G**	**R × G**
ASP	1.386 ± 0.107	1.720 ± 0.205	2.323 ± 0.491	2.096 ± 0.197	2.093 ± 0.271	2.136 ± 0.218	1.711 ± 0.137	0.162	0.100	0.174
SER	9.448 ± 0.645	10.117 ± 0.558	8.791 ± 0.846	11.961 ± 1.072	13.469 ± 1.238	11.398 ± 0.723	10.116 ± 0.528	0.801	0.005	0.152
GLU	0.789 ± 0.091	1.146 ± 0.088	1.176 ± 0.210	0.910 ± 0.086	1.255 ± 0.247	1.111 ± 0.113	0.999 ± 0.094	0.231	0.447	0.203
GLY	1.819 ± 0.073	1.899 ± 0.130	1.925 ± 0.075	1.949 ± 0.131	1.930 ± 0.092	1.829 ± 0.037	1.980 ± 0.080	0.850	0.063	0.003
HIS	5.138 ± 0.779	6.166 ± 1.272	3.531 ± 0.286	3.832 ± 0.312	5.493 ± 0.829	4.269 ± 0.248	5.395 ± 0.674	0.051	0.128	0.807
ARG	1.032 ± 0.055	1.071 ± 0.076	1.282 ± 0.055	1.109 ± 0.054	1.048 ± 0.075	1.112 ± 0.039	1.105 ± 0.046	0.078	0.892	0.683
THR	2.609 ± 0.079	2.650 ± 0.037	2.794 ± 0.116	2.833 ± 0.064	2.904 ± 0.087	2.763 ± 0.054	2.752 ± 0.054	0.104	0.887	0.944
ALA	9.346 ± 0.918	10.678 ± 1.284	7.060 ± 1.125	6.875 ± 0.736	8.502 ± 1.048	8.077 ± 0.687	8.908 ± 0.689	0.075	0.347	0.150
PRO	14.137 ± 0.860	14.693 ± 1.040	15.180 ± 0.382	12.724 ± 0.916	13.127 ± 0.709	13.512 ± 0.498	14.433 ± 0.546	0.224	0.264	0.806
GABA	41.459 ± 0.862 a^a^	37.442 ± 1.791 bc	40.536 ± 1.681 ab	41.644 ± 0.860 a	36.808 ± 1.286 c	40.177 ± 0.954	38.978 ± 0.863	0.012	0.305	0.852
CYS	0.044 ± 0.002 b	0.048 ± 0.003 b	0.053 ± 0.002 ab	0.092 ± 0.024 a	0.049 ± 0.006 ab	0.053 ± 0.003	0.061 ± 0.010	0.026	0.435	0.125
TYR	1.065 ± 0.065	0.926 ± 0.052	1.100 ± 0.079	1.183 ± 0.071	1.066 ± 0.071	1.057 ± 0.042	1.080 ± 0.047	0.109	0.703	0.563
VAL	3.135 ± 0.066	3.079 ± 0.152	3.558 ± 0.137	3.407 ± 0.086	3.355 ± 0.178	3.323 ± 0.085	3.290 ± 0.089	0.124	0.795	0.920
MET	0.513 ± 0.033	0.476 ± 0.034	0.566 ± 0.042	0.556 ± 0.033	0.527 ± 0.046	0.0519 ± 0.025	0.536 ± 0.023	0.480	0.609	0.130
LYS	3.453 ± 0.178 abc	3.121 ± 0.215 c	4.005 ± 0.228 a	3.753 ± 0.194 ab	3.377 ± 0.163 bc	3.583 ± 0.116	3.501 ± 0.150	0.024	0.601	0.721
ILEU	1.412 ± 0.058	1.360 ± 0.085	1.677 ± 0.098	1.613 ± 0.098	1.504 ± 0.076	1.517 ± 0.046	1.510 ± 0.066	0.060	0.893	0.947
LEU	2.187 ± 0.094 b	2.490 ± 0.250 b	3.306 ± 0.275 a	2.401 ± 0.178 b	2.421 ± 0.166 b	2.510 ± 0.115	2.612 ± 0.168	0.001	0.601	0.449
PHE	1.027 ± 0.040	0.918 ± 0.096	1.136 ± 0.053	1.064 ± 0.091	1.074 ± 0.123	1.056 ± 0.031	1.032 ± 0.071	0.409	0.678	0.615

a *Results are expressed as the mean ± SE. For each main effect, different lowercase letters in a row indicate a significant difference by the least significant difference test at P < 0.05*.

## Discussion

In general, the effects of rootstocks on the populations of aerial pests have been poorly studied. The first report on the influence of commercial rootstocks on the population density of the aerial pest *B. tabaci* in a hydroponic tomato crop was reported by Žanić et al. (2017). In the current study, an alternation of *B. tabaci* density was identified as a main effect of the rootstock cultivars (Tables 2, 3). The density of large nymphs and adults was higher on grafted treatments than on non-grafted (Tables 2, 3) as a result of lower attractiveness of non-grafted commercial rootstocks to *B. tabaci*. However, a significant interaction of cultivar x grafting confirmed the importance of rootstock and scion for large nymphs density (Figure 3). Thus, Clarabella grafted onto Arnold, Buffon, Emperador, or Maxifort reduced the incidence of *B. tabaci* large nymphs (Figure 3) compared with non-grafted and self-grafted Clarabella. The effects of grafting tomatoes onto wild *Solanum* spp. on the reduction of the incidence of *B. tabaci* adults was also reported by Alam et al. (1995), Alvarez-Hernandez et al. (2009), and Cortez-Madrigal (2010). Similarly, Žanić et al. (2017) found that tomato grafting onto Arnold and/or He-Man rootstocks could be valuable tool in an integrated management strategy against *B. tabaci*. The effects of grafting on other aerial pests have been investigated in several studies. Alvarez-Hernandez et al. (2009) and Cortez-Madrigal (2010) recorded that tomatoes grafted onto their wild relatives showed resistance to tomato psyllid *Bactericera* (= *Paratrioza*) *cockerelli* (Sulc) and aphids (Aphididae), in addition to the effect on *B. tabaci*. However, in a study on grafted potatoes, rootstocks did not affect the phloem-feeding potato aphid *Macrosiphum euphorbiae* Thomas but did reduce the development of the Colorado potato beetle (*Leptinotarsa decemlineata* Say) on potato scions (Pelletier and Clark, 2004). Furthermore, Lagenaria rootstock provided resistance to the mite *Tetranychus cinnabarinus* (Boisduval) in susceptible Cucurbita scions (Edelstein et al., 2000).

Although it has been suggested that grafting may improve resistance to aerial pests, these plant defense mechanisms have yet to be explored. According to Edelstein et al. (2000), resistance to *T. cinnabarinus* can be transferred by grafting from Lagenaria rootstocks to Cucurbita scions but not in the opposite direction. The authors suggested that the resistant rootstock produced a resistance-promoting substance that was transferred through the graft to the Cucurbita foliage. Cortez-Madrigal (2010) and Edelstein et al. (2000) suggested that both antixenosis and antibiosis mechanisms constitute possible types of resistance to foliar pests. Muigai et al. (2002) suggested the necessity of multifactorial approaches to explain resistance mechanisms among *Solanum* germplasm. To contribute to understanding of the mechanisms defining the attractiveness of plant to aerial pests, the leaf anatomy and the composition of amino acids of phloem sap were observed in our study.

In our experiment, the leaf anatomy characteristics of the tested rootstock cultivars and scion grafted onto rootstock varied significantly. Our results (Figures 5A,B) showed that grafting *per se* reduces leaf lamina and mesophyll thickness, as the thickness of both was lower in self-grafted Clarabella than in non-grafted Clarabella. However, the changes in leaf anatomy characteristics of Clarabella scion also depended on the rootstock cultivar. The leaf lamina and mesophyll thickness of Clarabella scion grafted onto Buffon and Maxifort was substantially less than that of non-grafted and self-grafted Clarabella; these thicknesses were reduced because of characteristics of the laminae and mesophyll of non-grafted Buffon and Maxifort rootstocks. The reduction in leaf lamina and mesophyll thickness was not so evident after grafting Clarabella onto Arnold and Emperador rootstocks, and the laminae were thicker than those of non-grafted Arnold and Emperador rootstocks. Buffon and Maxifort, both of whose vigor is less than that of Arnold and Emperador (personal observation), transferred their leaf properties to the scion, and the leaves remained as thin as those of non-grafted plants. The changes in leaf anatomy enhanced by grafting or by rootstock cultivar substantially influenced the attractiveness of the leaves to *B. tabaci* infestation and increased the population density of this insect. Non-grafted Clarabella plants were the most attractive for *B. tabaci* and in these plants, the leaf lamina thickness was 171.43 μm, the abaxial epidermis thickness was 12.09 μm, and the adaxial epidermis thickness was 17.37 μm, while the mesophyll thickness was 145.20 μm (Figure 5).

There is a lack of data on the effects of grafting on leaf anatomy with respect to defense mechanisms against whiteflies in vegetable crops. However, the leaf anatomy characteristics in plants preferred by whiteflies (vegetables or cotton) have been linked to pest densities. The leaf lamina thickness of eggplant varieties was positively correlated with the number of *B. tabaci* adults but not eggs (Hasanuzzaman et al., 2016). Eggplant leaves with thinner laminae were less succulent and less preferred for feeding and oviposition purposes by the whiteflies than were the leaves with thicker laminae. According to the study of Taggar and Gill (2012), the leaf laminae of black gram genotypes were positively correlated with the numbers of *B. tabaci* eggs, nymphs and adults. The genotypes with the thickest laminae had the largest nymphal populations. Venugopalrao et al. (1990) suggested a role of leaf lamina thickness in preventing the penetration of cotton plants by *B. tabaci* females because the plants the most susceptible to *B. tabaci* were those with the thinnest leaf laminae. On the other hand, Firdaus et al. (2011) indicated that pepper cuticle thickness was negatively correlated with *B. tabaci* adult survival and oviposition rates. Jauset et al. (1998, 2000) showed that the thickness of abaxial tomato cuticle, in combination with nitrogen level and water content, could also influence the survival of *T. vaporariorum*. In our study, the adaxial epidermis of Clarabella scion grafted onto Buffon, Emperador and Maxifort was similar to that of some non-grafted and self-grafted plants; only in Arnold the adaxial epidermis thickness was significantly reduced (Figures 5C,D). However, regarding the abaxial epidermis, only Clarabella grafted onto Emperador had a similar thickness as that of non-grafted and self-grafted Clarabella. The rootstock effect on Clarabella scion seems less clear for the thickness of abaxial and adaxial epidermis than for lamina and mesophyll thickness, and epidermis is not as clearly associated with *B. tabaci* infestation.

*Bemisia tabaci* adult and nymphal feeding behavior occurs via stylet penetration through the leaf laminae. Although several authors list *B. tabaci* (B biotype) as a phloem feeder (van Lenteren and Noldus, 1990; Byrne and Bellows, 1991), there is a report of possible alternative feeding methods (Cohen et al., 1996). It seems that *B. tabaci* may feed incidentally on non-vascular tissue, but at some point in their feeding the insects are obliged to tap into a vascular bundle (Cohen et al., 1996). In the study involving cotton plants (Cohen et al., 1996), the mean distance of the nymphs of *B. tabaci* from the point of labial contact with the leaf surface to the nearest edge of a vascular bundle was 40.9 μm. In the same plant species, the distance from the underleaf surface to the center of the nearest minor vascular bundle was significantly correlated with leaf thickness but negatively correlated with *B. tabaci* adult, egg and nymphal densities (Chu et al., 1999). In our study, the distance from the underleaf surface to the center of the nearest minor vascular bundle in non-grafted Clarabella plants, which were preferred the most by *B. tabaci*, was 52.54 μm (Figure 6). The longest distance was found for Arnold grafted using Clarabella as a scion, followed by self-grafted Clarabella and non-grafted Clarabella, and this distance coincided with lamina thickness. We consider that the distance of 52.54 μm from the underleaf surface to the center of the nearest minor vascular bundle in non-grafted Clarabella enables successful feeding of B. tabaci because of the highest pest density in this treatment.

In addition to changes in leaf anatomy, we expected that the tomato rootstocks or grafting could induce changes in plant metabolism by influencing the amino acid composition and thus affect feeding behavior and population density of *B. tabaci*. Eighteen amino acids were detected in the phloem sap exudate of Clarabella scion in our study, and the most abundant amino acid was GABA, followed by proline, serine, alanine, and histidine. In contrast to the results of a study by Valle et al. (1998), we detected a relatively small content of glutamic acid but more serine and alanine, as well as, the presence of proline and histidine. It has been proposed that GABA accumulation may be an artifact of the EDTA phloem exudation technique (Girousse et al., 1991) or may be caused by the wound response to the leaf cutting methodology used during EDTA-enhanced exudate collection (Valle et al., 1998). On the basis of experiments with *Choristoneura rosaceana* Harris larvae, Ramputh and Bown (1996) proposed that rapid GABA accumulation may represent one of many chemical defense systems that immobile plants deploy against phytophagous invertebrates. In soybean, the accumulation of GABA and reductions in glutamate are triggered by the mechanical damage of leaves, temperature changes, and darkness (Wallace et al., 1984). A high GABA content and low glutamic acid content were also observed in our study, probably because all treatments were infested by *B. tabaci* albeit to different extents. The GABA percentage in phloem sap exudates was dependent on rootstock and Buffon and Maxifort had lower contents of GABA than Arnold and Emperador but they were no less infested with *B. tabaci*. The scion cultivar Clarabella was the most attractive to *B. tabaci* and had a higher content of leucine than did rootstock cultivars, and a higher content of lysine compared to Buffon and Maxifort (Table 5). Increased levels of histidine, isoleucine, leucine, tyrosine and valine were found in tomato plants as result of interaction between mite *Tetranychus evansi* Baker & Pritchard infestation and drought (Ximénez-Embún et al., 2016), although the effect of drought was more pronounced.

In *Cucumis melo* L., quantitative and qualitative changes in amino acids did not cause variability in oviposition of *B. tabaci* biotype B, but higher adult (male and female) weight and developmental time are associated with higher concentrations of essential amino acids (Blackmer and Byrne, 1999). Grafting effects on the amino acid profile were observed only for lower contents of serine in grafted plants than in non-grafted plants. In a study by Bi et al. (2003), the levels of free amino acids in cotton petioles and the numbers of whiteflies (adults or immatures) were not correlated. However, the performance of the aphids *Myzus persicae* (Sulzer) and *Macrosiphum euphorbiae* (Thomas) could be linked to changes in the amino acid composition of the phloem; these changes included a developmental shift from high glutamine levels in pre-tuber-filling plants to low glutamine levels in tuber-filling plants (Karley et al., 2002). We did not find a simple relationship between free amino acid profiles in the phloem sap of tomato and *B. tabaci* density; however, the percentages of GABA, cysteine, lysine and leucine in the phloem sap depended on the rootstock, and serine was reduced by grafting. In plants infested by mites (Acarina, Tetranychidae) a composition of amino acids was related to host plant and cultivar (Dabrowski and Bielak, 1978). Herbivore arthropods are able to adopt on alternation in plant nutritional status and stress metabolites induced as a plant defense mechanism by changing enzyme system activity of the digestion system (Ortego, 2012; Zhu-Salzman and Zeng, 2015).

Our data reveal the *B. tabaci* density is affected by rootstock cultivar and grafting, as well as their interaction in the case of large nymphs. From a horticultural point of view, tomato grafting onto Arnold, Buffon, Emperador and Maxifort rootstocks can be a useful part of an integrated management strategy against this aerial pest. The underlying mechanism could be at least partly explained by changes in leaf anatomy, in which reductions in spongy parenchyma were observed after self-grafting or grafting onto rootstocks. Leaves with thinner laminae were generally less attractive to *B. tabaci*. The amino acid profiles shifted largely to the accumulation of GABA in all treatments and this may be due to the infestation of *B. tabaci* in all treatments. The scion cultivar Clarabella was the most attractive to *B. tabaci* and had a higher content of leucine than did rootstock cultivars, and a higher content of lysine compared to Buffon and Maxifort. However, a specific shift in free amino acids accumulation due to rootstock or grafting and its relation to *B. tabaci* density was not observed in this study. To distinguish the role of rootstocks in the amino acid profiles of scion, non-infested plants should be used as controls, as no simple response was observed in our study under biotic stress conditions.

## Author contributions

The author contributions were as follows: KŽ, GD, and SG conceived and designed the study; GD, MM, BU, GV, and KŽ conducted the experiments; KŽ and MM managed and evaluated the insect populations; IL, VB, GD, and SG measured the amino acid profiles; IB, GV, and BU performed the histology experiments; GD and SG analyzed the data; and KŽ drafted the manuscript. All authors read and approved the final manuscript.

### Conflict of interest statement

The authors declare that the research was conducted in the absence of any commercial or financial relationships that could be construed as a potential conflict of interest.
